# *Microcystis* Sp. Co-Producing Microcystin and Saxitoxin from Songkhla Lake Basin, Thailand

**DOI:** 10.3390/toxins13090631

**Published:** 2021-09-08

**Authors:** Ampapan Naknaen, Waraporn Ratsameepakai, Oramas Suttinun, Yaowapa Sukpondma, Eakalak Khan, Rattanaruji Pomwised

**Affiliations:** 1 Environmental Assessment and Technology for Hazardous Waste Management Research Center, Faculty of Environmental Management, Prince of Songkla University, Hat Yai 90110, Thailand; ampapan.nk@gmail.com (A.N.); oramas.s@psu.ac.th (O.S.); 2 Office of Scientific Instrument and Testing, Prince of Songkla University, Hat Yai 90110, Thailand; waraporn.ra@psu.ac.th; 3 Center of Excellence on Hazardous Substance Management (HSM), Bangkok 10330, Thailand; 4 Division of Physical Science, Faculty of Science, Prince of Songkla University, Hat Yai 90110, Thailand; yaowapa.suk@psu.ac.th; 5 Department of Civil and Environmental Engineering and Construction, University of Nevada, Las Vegas, NV 89154-4015, USA; eakalak.khan@unlv.edu; 6 Division of Biological Science, Faculty of Science, Prince of Songkla University, Hat Yai 90110, Thailand

**Keywords:** Songkhla Lake Basin, *Microcystis*, extracellular polymeric substances, microcystins, saxitoxin

## Abstract

The Songkhla Lake Basin (SLB) located in Southern Thailand, has been increasingly polluted by urban and industrial wastewater, while the lake water has been intensively used. Here, we aimed to investigate cyanobacteria and cyanotoxins in the SLB. Ten cyanobacteria isolates were identified as *Microcystis* genus based on16S rDNA analysis. All isolates harbored microcystin genes, while five of them carried saxitoxin genes. On day 15 of culturing, the specific growth rate and *Chl-a* content were 0.2–0.3 per day and 4 µg/mL. The total extracellular polymeric substances (EPS) content was 0.37–0.49 µg/mL. The concentration of soluble EPS (sEPS) was 2 times higher than that of bound EPS (bEPS). The protein proportion in both sEPS and bEPS was higher than the carbohydrate proportion. The average of intracellular microcystins (IMCs) was 0.47 pg/cell on day 15 of culturing, while extracellular microcystins (EMCs) were undetectable. The IMCs were dramatically produced at the exponential phase, followed by EMCs release at the late exponential phase. On day 30, the total microcystins (MCs) production reached 2.67 pg/cell. Based on liquid chromatograph-quadrupole time-of-flight mass spectrometry, three new MCs variants were proposed. This study is the first report of both decarbamoylsaxitoxin (dcSTX) and new MCs congeners synthesized by *Microcystis*.

## 1. Introduction

The overgrowth of cyanobacteria leads to critical environmental problems and ecological events, including hypoxia, reducing water clarity, and imbalance of food webs. Among bloom-forming cyanobacteria, *Microcystis* sp. are the most abundantly reported [1]. The organisms are capable of producing several toxins, including hepatotoxic microcystins (MCs) [2] and neurotoxins (anatoxin-a, cyanopeptolin, and β-N-methylamino-L-alanine) [3]. MCs cause liver failure in humans and animals and increase the risks of primary liver cancer in humans [4]. Co-production of MCs and other toxins has been reported in certain *Microcystis* spp. *Microcystis* producing both MCs and saxitoxins (STXs) were isolated from a Brazilian reservoir [5] and freshwaters in Scotland and Brazil [6]. Saxitoxins, comprising 57 compounds, are a potent neurotoxin blocking ionic sodium and calcium channels, resulting in rapid paralysis and respiratory failure [7]. The passing of either MCs and/or STXs through the aquatic food web leads to animal deaths and human poisonings [8,9]. The World Health Organization (WHO) and the United States Environmental Protection Agency (EPA) have set guidelines to minimize MCs’ hazards [10,11]. The drinking water guidelines are 0.3 µg/L (bottle-fed infants and pre-school children) and 1.6 µg/L (school-age children and adults) MCs. The cyanobacterial guidelines for recreational water have been set as low effect (<20,000 cells/mL), moderate effect (20,000–100,000 cells/mL), and high effect (100,000–10,000,000 cells/mL). An official guideline for STXs has been established in two states in the U.S. (0.2 µg/L in Ohio and 0.3 µg/L in Oregon), and there is an Australia drinking water guideline (3 mg/L).

The success of a *Microcystis* bloom is attributed to its ability to migrate rapidly and defend against predation via colony formation and aggregation [12]. Extracellular polysaccharide (EPS) plays a critical role in colony formation. EPS also facilitates aggregated cyanobacteria floating near the water surface, known as scum formation [13]. The EPS composition and structure contribute to its matrix stability [14]. Its constituents (polysaccharide, protein, lipids, and nucleic acid) are diverse among *Microcystis* species [15].

*Microcystis* blooms have predominantly occurred in freshwater when the temperature rises [16]. *Microcystis*-harboring salt-tolerant genes has been reported in estuaries and marine environments [2,17,18]. The accumulation and biomass are facilitated by carbon fixation, availability of macronutrient nitrogen (N) and phosphorus (P), and physical parameters [19]. Increasing N supply would promote *Microcystis* sp. population [20,21] and increased MCs production [22].

Songkhla Lake Basin (SLB), a total area of 986.8 km^2^, is the largest natural lake in Thailand and Southeast Asia. The SLB is a unique ecosystem, which combines freshwater, brackish water, and saline water. The impurity in SLB originated from urbanization, industrialization, and artisanal fishing since along the shore are urban settlements, fishing villages, shrimp ponds, seasonally flooded forests, and rice paddy fields. Biological studies of the SLB have concentrated primarily on the taxonomy and biology of a few economically important species [23,24,25]. The presence of chlorophyll a (*Chl-a*) content has been suggested as a reason for phytoplankton propagation, including cyanobacteria in water bodies [26]. Algal blooms have occurred in SLB periodically, especially after rainy seasons [27]. The Marine and Coastal Resources Research Center, Lower Gulf of Thailand, revealed *Chroococcus*, *Merismopedia*, *Microcystis*, *Oscillatoria*, and *Spirulina* in SLB without algal blooming. While nutrient loading into SLB tends to increase dramatically, studies of harmful cyanobloom conditions in the lake have been limited by a lack of contaminated cyanotoxin and toxic *Microcystis* reports.

Our study provides proper identification of taxa and an evaluation of cyanotoxin accumulation in SLB for setting sound management procedures to reduce/prevent the exposure risk. Here, we aimed to investigate water parameters and toxin-producing *Microcystis* in several locations around the SLB. The toxin production and release were also determined as aquatic animal and human health benefits. This study aimed to indicate the potential harmful cyanobacteria in this unique environment and contribute to the discovery of novel toxic compounds.

## 2. Results

### 2.1. General Water Quality Parameters and Bacterial Isolation

**Figure 1:**
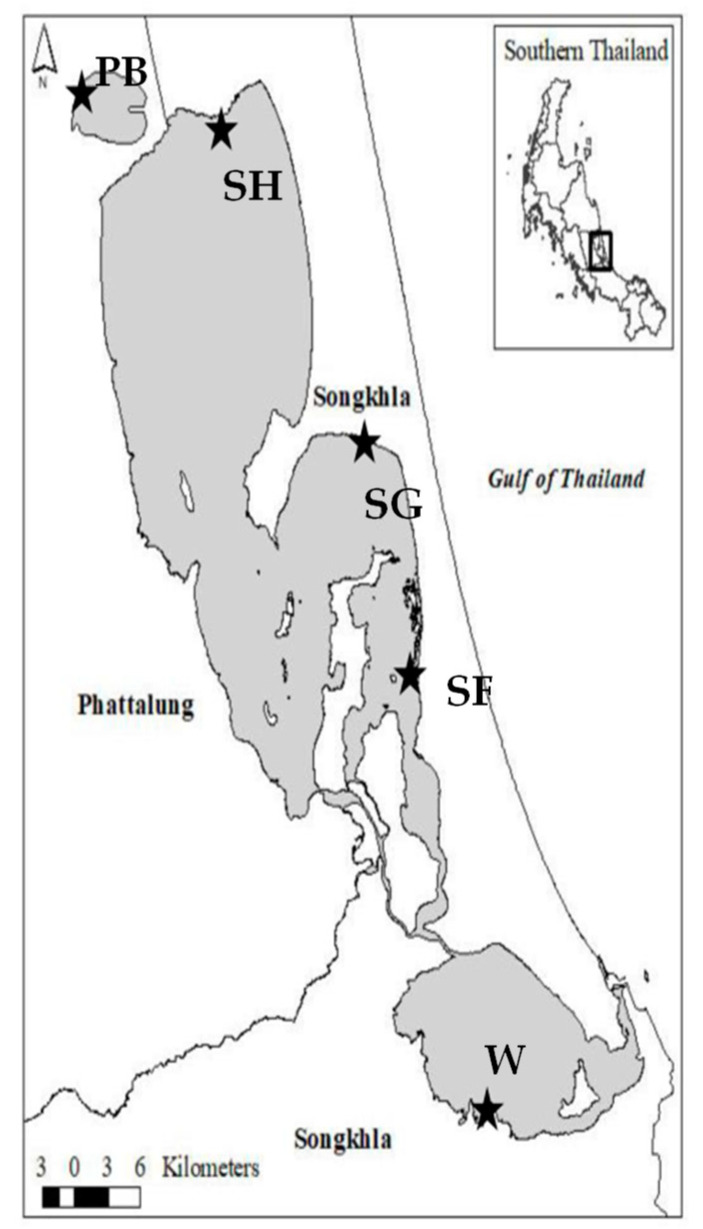
Map of Songkhla Lake. The map shows the locations of water sampling. PB = Thale Noi, Pattalung, Thailand; SF, SG, and SH = Songkhla Lake, Songkhla; W = Hat Yai municipal wastewater treatment plant.

A total of five water samples were obtained and used in this study. Four water samples were collected along accessible shores of Songkhla Lake Basin (SLB) (Figure 1). The sample sites are located close to urban settlements. One wastewater sample was collected from Hat Yai municipal wastewater treatment plant (HMWTP), which was effluent water drained to SLB (Figure 1). All samples were collected in January, the end of the rainy season (September–December). Nutrients easily load and accumulate in the lake, favoring cyanobacterial proliferation [28]. For all samples, biochemical oxygen demand (BOD) and chemical oxygen demand (COD) were 1.58–5.4 mg/L and 28–50 mg/L, respectively (Table 1). Total phosphorous and total nitrogen were 0.08–0.24 mg/L and 0.58–1.15 mg/L, respectively. Other water quality characteristics included dissolved oxygen (DO) of 1.82–7.02 mg/L, pH of 6.7–8.34, and salinity of 0.1–0.3 g/L. The lowest DO level (1.82 mg/L) that appeared at SG might have resulted from restricted water exchange, wind calm, and high tidewater. Salinity (0.3 g/L) indicated that two water samples from Songkhla Lake, Songkhla, were brackish. The onsite temperature of the samples ranged from 29 to 32 °C.

**Figure 2:**
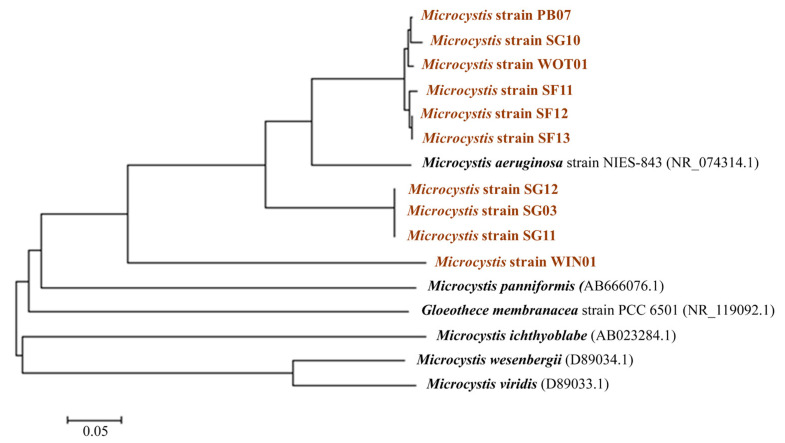
Phylogenetic tree based on 16S rDNA gene sequences. The bar scale indicates 5% divergence.

Cyanobacteria were isolated from the water samples. Ten green colonies were selected and observed under an optical microscope. The cells ranged from 1 to 5 µm in diameter with a round shape and green color. The 16S rDNA analysis indicated that all strains fell into Microcystaceae family and *Microcystis* genus. The phylogeny based on 16S rDNA sequences was generated by the neighbor-joining tree method; compared with *M. aeruginosa*, *M. panniformis*, *M. ichthyoblabe*, *M. wesenbergii*, *M. viridis*, and *Gloeothece membranacea*; and deposited in the NCBI database (Figure 2). Most *Microcystis* isolates were closely related to *M. aeruginosa* strains NIES-843 (NR_074314.1), except WIN01. SG12, SG03, and SG11 isolated from the exact location were in the same phylogenic group. SH13, SH12, and SF11 also showed similar 16S rDNA gene sequences. Three *Microcystis* strains isolated from Songkhla Lake (SG10), Thale Noi (PB07), and HMWTP (WOT01) fell into the same cluster. Our results reveal that despite the absence of cyanobacterial bloom, *Microcystis* was detected. Cyanoblooom is facilitated by other environmental factors, including nutrients, carbon dioxide, weather conditions, water bodies, salinity, heavy metals, and sunlight [29,30].

### 2.2. Microcystin Genes and a Saxitoxin Gene Analysis

Microcystin genes and a saxitoxin gene were investigated in all 10 strains. The MC gene cluster is arranged in two divergently transcribed operons (ORFs), *mcy* A-C and *mcy* D-J. The *mcy* A-C operon is engaged in synthesizing amino acids attached to Adda through a subsequent modification or tailoring by *mcyJ*, *mcyE*, and *mcyF* genes [31]. The *mcyA* genes are widely employed to detect the toxigenic *Microcystis* genus [32,33,34]. Our results show that all isolates carried *mcyA* (Table 2). The *mcyA^a^* primers detected the target gene in 5 out of 10 strains, while the *mcyA* gene was detected in 9 of 10 strains by *mcyA^b^* primers. The *mcyA^b^* primers were created based on nucleotides of toxigenic *Microcystis* and *Planktothrix* strains [35]. However, certain non-toxic strains harbor the *mcy* genes [36,37]. Our study suggests that the *mcyA* primers might be restricted by a small nucleotide database of the toxic strains and that *mcyA* sequences are divergent among *Microcystis* isolates [32]. A report on MCs detection in Greece revealed that despite the presence of the toxin, the *mcyA* gene was not detected in freshwater samples [38]. Therefore, MCs prediction strictly based on molecular assay might be insufficient. Combining molecular techniques (PCR) and other techniques such as chemical methods (HPLC) and immunoassay will assure the MC presence.

Our results show that 5 of 10 strains were positive for *sxtA* and also carried *mcyA* (Table 2). The STXs biosynthesis initiates via PKSs-like enzymes encoded by *sxtA* [39]. The *sxtA* is a core gene among the gene clusters for the synthesis of STX and analogs [40,41]. This gene has been widely used to detect STX producers [42].

### 2.3. Growth and EPS Composition

The *Microcystis* density and *Chl-a* content were measured. The specific growth rate of all isolates was 0.2–0.3 day^−1^, as shown in Table 2. As a general metric for algal biomass, *Chl-a* was employed as a reference for cyanoblooms [43]. Our results show that *Chl-a* production was consistent with bacterial growth. *Chl-a* was 0.1 to 4 µg/mL from day 0 to day 15.

Extracellular polymeric substance (EPS) of *Microcystis* is produced as part of the metabolism process. It forms and surrounds the cell surface as a protective barrier between the cell and the external environment [12,44,45]. The content, types, and composition of EPS also facilitate colony formation, impacting the distribution and persistence of cyanobacteria [46,47,48]. A study by Holland et al. suggested that *Microcystis* with increased colony size contained higher MCs [49]. Therefore, correlations between MCs and the content and composition of EPS are needed to be revealed.

**Figure 3:**
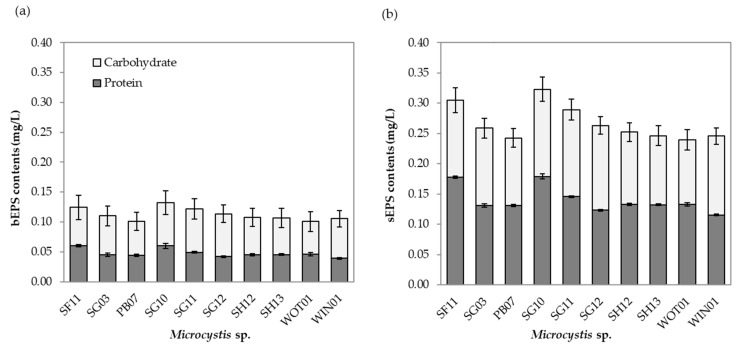
Protein and carbohydrate content of bEPS (**a**) and sEPS (**b**) after 15 days of incubation. The data show the means ± standard deviations based on triplicated experiments.

Here, we determined EPS content and composition in 10 *Microcystis* isolates (Figure 3). The total EPS varied from 0.37 to 0.49 µg/mL. The highest total EPS concentration was found in SG10. For all isolates, the average soluble EPS (sEPS) concentration (Figure 3b) was roughly twice higher than the average bound EPS (bEPS) concentration (Figure 3a) (0.27 vs. 0.14 µg/mL). Our results confirm the finding by Xiao et al. (2018), that *Microcystis* are commonly unicellular cells under an axenic condition [12]. High sEPS content was found in unicellular cells, while increasing bEPS content could be concluded for colony formation [46]. The proportion between protein and carbohydrate in sEPS in 10 isolates was similar. In contrast, the protein proportion in bEPS was higher, ranging from 2.014 to 3.056 at an average value of 2.369, which was in agreement with a previous study [50]. However, the proportions of bEPS in this study were inconsistent with Xu et al. (2013), which revealed a higher polysaccharides content than protein. The EPS composition also depends on cyanobacterial species, including *Synechocystis* sp. (FACHB898), *Synechococcus* 7942 (FACHB805), *Microcystis flosaquae* (FACHB1028), *Scytonema hofmanni* (FACHB 248), and *Nostoc* sp. (FACHB106) [51].

During cyanobloom, large aggregated colonies form scum floating on a water body’s surface [52]. This upward cell migration is enhanced by photosynthetic activity resulting in O_2_ saturation and nucleation into bubbles [13,53] and gas vesicles in *Microcystis* cells [47]. The O_2_ bubble is trapped inside EPS, indicating that scum formation is highly correlated with the amount of EPS production [13,54]. Here, the scum formation was enhanced by culturing in high concentration media and was found in SG03, SG11, and SH13 after culturing for 2 to 7 days, in agreement with previous studies [13,53]. The remaining isolates did not generate scums over the incubation period. Future work should be conducted to determine the effects of EPSs composition on scum formation and large aggregated colonies.

### 2.4. Microcystins Production of Microcystis *Sp.*

**Figure 4:**
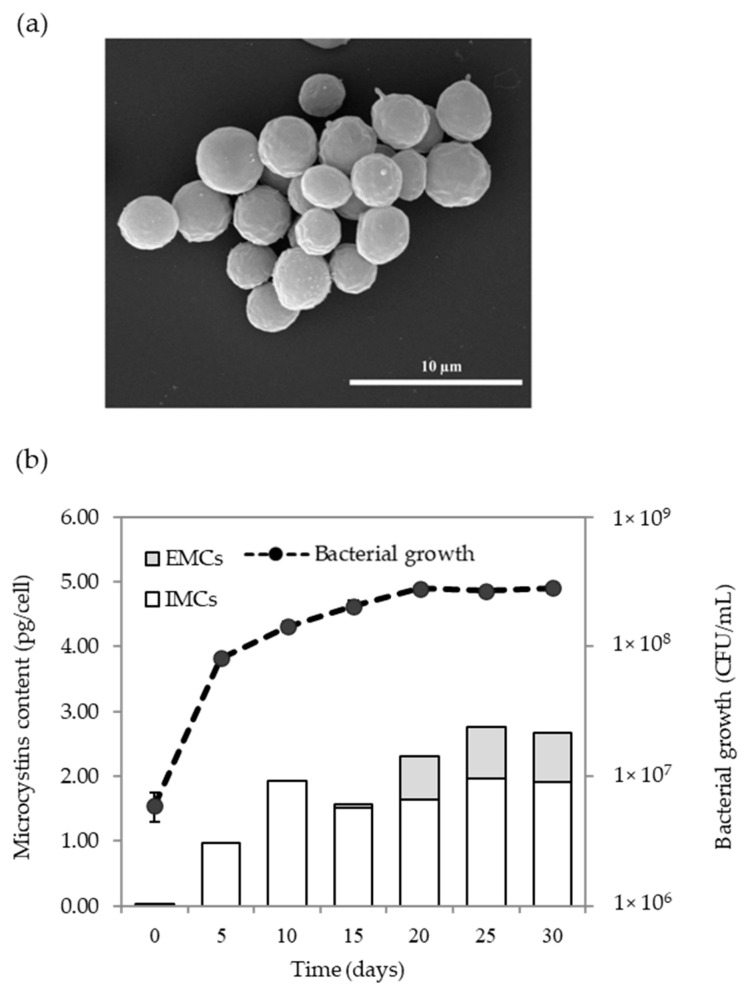
A 30-day axenic culture of *Microcystis* SG03; (**a**) SEM image of *Microcystis* cells at 10 days and (**b**) MCs production and growth of SG03.

After 15 days of culturing intracellular microcystins (IMCs) and extracellular microcystins (EMCs), production in all *Microcystis* isolates was determined by ELISA. This technique is recommended to quantify several MCs variants in cyanobacterial cells and to track MCs production in water bodies [55,56]. The test kit is designed to detect Adda, which is a non-protein amino acid at position 5 in MCs, with cross-reactivity between different MCs variants [57]. Our results show that high IMCs production ranging from 210 to 314 µg/mL was observed in *Microcystis* isolated from Songkhla Lake. *Microcystis* sp. isolated from HMWTP was approximately 280 µg/mL of MCs (Table 2). The EMCs were not detected in any isolates. The absence of EMCs might be the result of low cell density after 15 days of culturing (10^7^ CFU/mL) and the kit’s sensitivity. More prolonged cultivation than 15 days resulted in EMCs production (Figure 4b). SG03 produced IMCs at the highest concentration of 1.3 pg/cell, while SH13 was the lowest in IMCs production at 0.14 pg/cell. *Microcystis* SG03 was observed under SEM. As seen in Figure 4a, with round shape, cell diameter ranged from 3 to 5 µm.

### 2.5. Kinetics of MCs Production Based on Microcystis Growth

Growth and MCs kinetics of SG03 were investigated from day 0 to day 30 (Figure 4b). The initial IMCs content at 0.03 pg/cell on day 0 was detected in the inoculum (6 × 10^6^ CFU/mL). On day 5, the specific growth rate (µ) based on cell number was 0.522 day^−1^, indicating that the exponential growth phase followed the late exponential growth phase from day 5 to 15 (µ = 0.186 day^−1^). The stationary growth phase spanned from day 20 to day 30 (µ = 0.002 day^−1^). MCs production was determined along the growth curve. The IMCs were dramatically produced at 1.94 pg/cell during the exponential growth phase (day 10), and then the production was steady when cyanobacteria entered the stationary growth phase (Figure 4b). The EMC production (0.06 pg/cell) was measured in the late exponential growth phase and dramatically rose on day 20 (0.66 pg/cell) during stationary growth, indicating cell death. Our results suggest that the MCs were the primary metabolite produced intracellularly at the log phase. Our finding is well supported by previous work in which the maximum MCs production of *Microcystis* was found in the log phase and persisted for months [43,58,59]. Studies have shown that MCs production rescues the cells from damage by photosynthesis, reactive oxygen species [60], and imbalance of C:N by redirecting primary metabolism in cells [61].

### 2.6. Identification of Cyanotoxins

**Figure 5:**
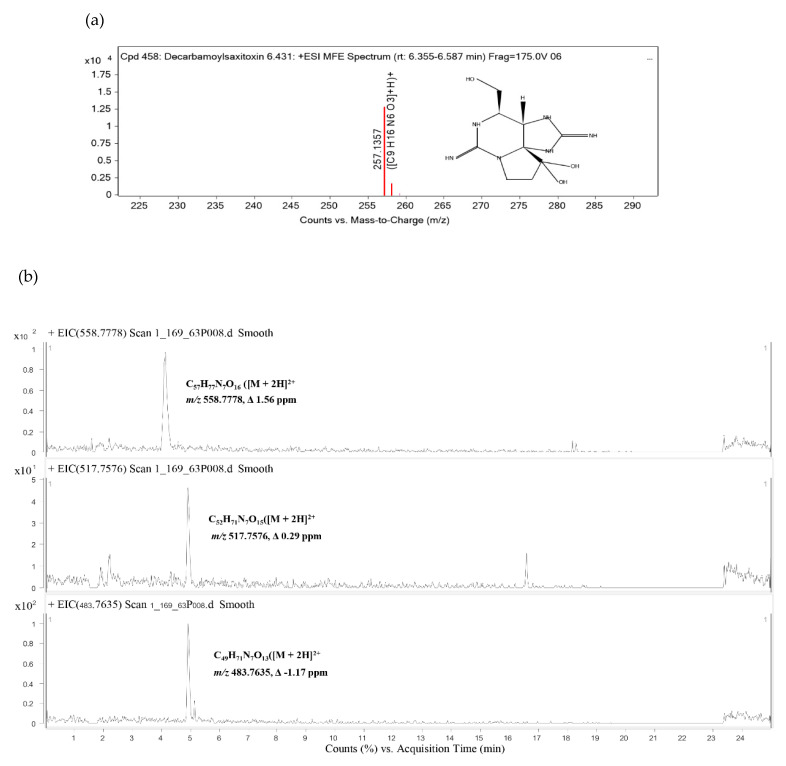
LC-QTOF MS spectrum (**a**) decarbamoylsaxitoxin, (**b**) tentative microcystins.

The cyanotoxin profiles of SG03 were investigated by LC-QTOF MS. Here, the emergence of both MCs and decarbamoylsaxitoxin (dcSTX)-producing *Microcystis* SG03 is reported (Table 3). At 14.579 min of the retention time, compound 1 was detected with *m/z* 257.1357, Δ −0.63 ppm. This compound was annotated as dcSTX with a score of 85% (Table 3; Figure 5a). The other three compounds were previously unreported MCs based on their probable chemical composition/structure and mass properties [62]. As shown in Figure 5b, they were: C_57_H_77_N_7_O_16_ ([M + 2H]^2+^ mass 1115.5409, Δ 1.56 ppm); C_52_H_71_N_7_O_15_([M + 2H]^2+^ mass 1033.5005, Δ 0.29 ppm); and C_49_H_71_N_7_O_13_([M + 2H]^2+^ mass 965.5125, Δ −1.17 ppm); designated as compounds 2, 3, and 4, respectively. The mass accuracy tolerances (Δ < 5 ppm) between found mass and calculated mass of compounds 2, 3, and 4 were Δ 1.61 ppm, Δ 0.29 ppm, and Δ −1.55 ppm, respectively.

## 3. Discussion

*Microcystis* blooms cause ecosystem loss as well as threats to public health and animals. *Microcystis* possesses a well-known hepatotoxin, MCs; a few strains also produce neurotoxin (β-N-methylamino L-alanine and paralytic shellfish poison) [63]. However, no STX biosynthesis genes have been identified in *Microcystis*.

Various factors facilitate the abundance of cyanobacteria in aquatic environments. EPSs prolong cyanobacterial persistence in water and protect cells from the environment. Cyanobacterial aggregation and scum formation can be promoted by higher EPS content [13,47,64]. The EPS composition involves colony size and colony formation [12,65]. *Microcystis* with increased colony size was reported to produce higher MCs [49]. Our results show that the average sEPS concentration was higher than the average bEPS concentration as a unicellular cell under an axenic condition. There was no statistical difference between EPS content and composition among all isolates. Therefore, correlations between MCs and EPS content and composition cannot be defined in this study. Associations of MCs and EPS types, content, and composition still need to be discovered.

The MCs can contaminate different environmental compartments, including sediment, animals, and aquatic and terrestrial plants [66]. MCs produced by *Microcystis* could persist for months in axenic cultures [67]. In our study, IMC production of *Microcystis* SG03 remained constant after 20 days of incubation. The averaged MCs production was 1.91 pg/cell on day 30 of culturing, with stable cell density. Under low nutrient/starvation conditions with sufficient light, the SG03 cells entered the stationary phase without changing MCs production. However, other studies reported increasing MCs production under nutrient starvation [68,69]. During stress conditions, MCs synthesis is enhanced by phycobilisomes degradation and by the toxins combined with phycobilins, stabilizing cell integrity [68,70]. Furthermore, MCs might protect the cells from oxidative stress under insufficient nutrients [71]. Our study indicates that *Microcystis* can produce MCs and survive for a long time, suggesting a need for a vigilant surveillance program on toxigenic *Microcystis* and MCs in aquatic areas.

To date, at least 279 MCs have been reported with various formulas: C_44–47_ H_63–84_ N_7–13_ O_12–17_ [72,73]. MCs structures are composed of cyclic oligopeptides consisting of seven amino acids, including D–Ala^1^–X^2^–D–MeAsp^3^–Z^4^–Adda^5^–D–Glu^6^–Mdha^7^ [31]. The X and Z are variable L amino acids. Adda is a 3-amino-9-methoxy-2, 6,8-trimethyl-10-phenyl-deca-4,6-dienoic acid, and Mdha is N-methyldehydroalanine. In our study, two compounds (2 and 3) showed characteristics of MCs containing Adda at position 5, while compound 4 consisted of DMAdda (Table 3 and Figure 5). D-Ala is mostly found in position 1 [72], consistent with compound 4. Position 1 of compounds 2 and 3 contained Gly and L-Ser, which have been found in MCs produced by *Nostoc* sp. and cyanobacterial mat samples [74,75]. In position 3 of MCs, D-erythro-β-methyl isoaspartic acid (D-MeAsp) and D-erythro-isoaspartic acid (D-Asp) are conserved with approximately 56% and 44%, respectively [72]. Our study shows that D-Asp was presented in compounds 2 and 3. Another highly conserved amino acid is D-Glu at position 6 [31]. The D-Glu could become Glu(OMe) from an extraction step through methanolic solutions [76,77,78]. Here, Glu(OMe) was found in compounds 2 and 4. The amino acid residue in position 7 is variable with commonly found N-methyldehydroalanine (Mdha). The Mdhb existed in compound 2, while compound 4 possessed tentative Mala. Even though several MC congeners have been presented in the environment and laboratories, their toxicity has been inadequately assessed.

Dominant STXs producers are filamentous cyanobacteria, including *Anabaena*, *Aphanizomenon*, *Cylindrospermopsis, Lyngbya*, *Phormidium*, *Planktothrix*, *Raphidiopsis*, *Scytonema*, and *Woronichinia* [79,80,81,82,83,84,85]. Only three unicellular *Microcystis* isolates were reported to produce STXs [5,6]. One of them co-produced mono-sulfated STX (GTX 1-4) and [L-ser^7^] MC-RR [5]. The other two isolates from eutrophic lakes (Arresø, Bagsværd Sø and Lyngby Sø) and mesotrophic lakes (Furesø and Esrum Sø) in Denmark produced STX without MCs production information [6]. Our study is the second to report the co-production of MCs and STX (by SG03). However, we, for the first time, discovered that *Microcystis* also synthesized dcSTX. Currently, 57 natural STX analogs have been identified [41]. The toxin divergence depends on PST-transforming enzymes between toxigenic organisms and contaminated bivalves [86]. Carbamoyl groups of the STXs are decarbamoylated, resulting in the presence of dcSTX. The enzymatic decarbamoylation of STX was a major presence in mussels and human metabolically active tissues [86,87].

The SLB has a unique ecosystem as fresh, brackish, and saline water lead to plenty of bioresources and high biodiversity. This area has benefited aquaculture, agriculture, factories, tourist resorts, and housing developments. Here, we isolated toxigenic *Microcystis*, which produced both MCs and STXs, from SLB with no observed blooms. Christensen et al. (2019) reported the production of both toxins before visible blooms [88]. When season changes combine with other environmental factors, harmful cyan blooms occur. In Thailand, MC accumulation was found in prawn farms [89,90], fish ponds [90,91,92], and recreational reservoirs [93,94], especially in the temperate region of the country with high N and P concentrations. In contrast, the reports of STX from Thailand were associated with only freshwater puffers [95,96]. The observation of MCs- and STXs-producing cyanobacteria in our study calls for more research on harmful cyanobacterial communities and toxin distribution in recreation areas and aquaculture farms in Songkhla Lake.

## 4. Conclusions

This is the first report of *Microcystis* in Songkhla Lake, Thailand. In axenic culture, *Microcystis* produced sEPS 2 times more than bEPS. The ratio of protein and carbohydrate was equal in sEPS, while the protein fraction was higher than carbohydrate in bEPS. All isolates produced MCs. One isolate (SG03) presented both MCs and dsSTX. SG03 produced three new tentative MC variants as undescribed congeners. The MCs accumulation in SG03 was observed until 30 days of incubation, and the toxin was released extracellularly on day 15. The IMCs were synthesized mainly in the middle exponential growth phase, while the EMCs were initially produced at the stationary growth phase. The rising of total MCs synthesis was observed during the exponential growth phase and was constantly produced during the stationary growth phase. This study indicates that harmful cyanobacteria could present in water bodies without visible blooms. The unique complexity of SLB provided groups of *Microcystis* producing novel toxic compounds. We exhibited that *Microcystis* extended some distance into brackish and/or marine water bodies. Further studies should focus on the effects of water quality parameters, including N and P on *Microcystis* aggregation, scum formation, and toxin production, thereby preventing harmful effects of cyanobacteria and their toxins.

## 5. Materials and Methods

### 5.1. Water Quality Characterization

Four lake water samples from Songkhla Lake Basin (SLB), Thailand (7687 km^2^ of land area and 1042 km^2^ of the Lake surface), were collected on 25 January 2019, without visible blooms (Figure 1). These locations were located close to urban settlements. Effluent water drained to the SLB was collected from the Hat Yai municipal wastewater treatment plant (HMWTP) on 25 January 2019 (Figure 1). The 5 samples containing 1 L with duplicates were taken at a 50 cm depth and kept on ice during transportation. The water quality, including biochemical oxygen demand (BOD), chemical oxygen demand (COD), total phosphorus (TP), total Kjeldahl nitrogen (TKN), temperature, pH, and salinity was characterized. All samples were used for *Microcystis* isolation in further experiments.

### 5.2. Microcystis *Spp.* Isolation

Ten milliliters of water samples and the duplicates were enriched in 10 ml of 2 × BG-11 broth (pH 7.5) [97]. The samples were incubated under a 12:12 h light/dark cycle (25 µmol photon m^−2^/s, cool fluorescent light) at 25 °C for 14 days and hand-shaken three times a day. The enriched samples were then streaked onto BG-11 agar plates supplemented with imipenem (10 µg/mL) [97], followed by incubation for 7–14 days under the above conditions. The isolated green bacterial colonies were obtained, and their morphology was documented. Characteristics of the cyanobacterial cells were observed under an optical microscope.

### 5.3. Cyanobacterial DNA Extraction

All cyanobacterial isolates were cultivated in 50 mL BG-11 under the above conditions for 10 days. Then, DNA from each isolate was extracted. The DNA extraction method was modified from Hisbergues et al. (2003) and Rogers and Bendich (1989). Briefly, the culture was centrifuged at 10,000× *g* for 5 min, and the pellet was resuspended in 300 µL suspension buffer (50 mM Tris-HCL (pH 8), 5 mM EDTA (pH 8), and 50 mM NaCl), followed by the addition of lysozyme (2 mg/L) and proteinase K (2 mg/mL). The cell suspension was incubated at 37 °C for 30 min and at 60 °C for 10 min. The mixture was added with an equal volume of lysis buffer (2% CTAB, 2% SDS, 1.4 M NaCl, 20 mM EDTA (pH 8), 100 mM Tris-HCl (pH 8), 2% 2-mercaptoethanol) and incubated at 70 °C for 1 h. The DNA was separated by adding an equal volume of phenol/chloroform/isoamyl alcohol (25:24:1, *v*/*v*), followed by centrifugation at 10,000× *g* for 15 min. The supernatant was collected and mixed with an equal volume of chloroform/isoamyl alcohol (24:1, *v*/*v*). Then, the mixture was centrifuged at 10,000× *g* for 15 min. The supernatant containing DNA was precipitated with 0.3 volume of 3 M sodium acetate and 1 volume of isopropanol and kept at −20 °C for 2 h. After centrifugation at 10,000× *g* for 20 min, the DNA pellet was obtained and washed with 1 mL of 70% ethanol. The pellet was air-dried and dissolved with 30 µL sterilized deionized water and kept at −20 °C.

### 5.4. Detection of Microcystin Genes, Saxitoxin Gene and 16S rDNA by PCR

Microcystin gene (*mcyA*), saxitoxin gene (*stxA*) [98], and 16S rDNA were investigated by PCR. Two sets of *mcyA* primers were applied: *mcyA^a^* primers [35] and *mcyA^b^* primers [32]. PCR amplifications were performed using a 25 µL mixture of 5 µL buffer (dNTP, Mg^2+^) (Bioline Reagents Ltd, London, UK), 3 µL each of primer-F and primer-R (1 µM), 0.25 µL MyTaq polymerase (1 unit), 1 µL DNA template (20 ng/mL), and 12.75 µL deionized water. The PCR thermal cycling programs included initial denaturation at 94 °C for 2 min, followed by 30 cycles at 94 °C for 30 s, annealing for 30 s, and extension at 72 °C for 1 min, with a final extension at 72 °C for 10 min. The PCR products were observed by electrophoresis on 1.5% agarose gels in 0.5 TBE buffer at 100 V for 40 min, with 1 kb DNA Ladder (Solis BioDyne) as the molecular-sized marker. The agarose gel was stained with ethidium bromide and imaged under a benchtop UV transilluminator (LUMIstar^®^ Omega. Ortenberg, Germany).

Cyanobacterial identification was based on 16S rDNA sequencing. Briefly, the 16S rDNA was amplified using the primer 27F and 809R [99]. The PCR product was purified using a GenepHlow^TM^ Gel/PCR kit and sequenced (Novogene co. Ltd., Cambridge, UK). Sequences were aligned using ClustalW followed by comparing their similarity with the other sequences in the Genbank database. A phylogenetic tree was constructed via a neighbor-joining (NJ) method by MEGA 7.0 software with 1000 replicates of bootstrap.

### 5.5. Measurements of Specific Growth Rate and Chlorophyll a (Chl-a)

The cyanobacteria isolate was transferred into 100 mL of BG-11 medium and adjusted to 10^6^ CFU/mL. The culture was incubated under a 12:12 h light/dark cycle (25 µmol photon m^−2^/s) at 25 °C for 15 days. Eleven milliliters were collected at three-day intervals.

To measure the cyanobacterial growth, 10 µL of each sample was dropped on a hemocytometer, and cell numbers were counted under a microscope. The specific growth rate (μ) was calculated from

μ = (ln N_2_ − ln N_1_) × (*t*_2_ − *t*_1_)^−1^ (1)

where N_1_ and N_2_ are the cell numbers at times *t*_1_ and *t*_2_, respectively.

To evaluate *chl-a* content, 10 mL of the culture was centrifuged at 3600× *g* for 10 min. The pellets were resuspended in 400 µL of acetone (90%, *v*/*v*) and kept at 4 °C for 24 h in darkness. After centrifugation at 3600× *g* for 30 min, the supernatant was measured for optical density (OD) using a UV–vis spectrophotometer (LUMIstar^®^ Omega, Ortenberg, Germany) at 630, 645, 663, and 750 nm, and 90% acetone solution was used as a blank [100]. The concentration of *Chl-a* was calculated as

*Chl-a* (μg/mL) = [11.64 × (OD_663_ − OD_750_)) − 2.16 × (OD_645_ − OD_750_) + 10 × (OD_630_ − OD_750_)] × 25 (2)

### 5.6. Microcystins Production

To measure the MCs, 10 *Microcystis* isolates were grown in 30 mL of BG-11 medium for 15 days, as described above. The culture was centrifuged at 6000× *g* for 10 min. The cell pellet and supernatant were collected to measure intracellular MCs (IMCs) and extracellular MCs (EMCs), respectively. The pellet was resuspended in 10 mL 50% methanol–water solution in order to extract IMCs [101]. Then cell suspension in the methanol solution was sonicated for 10 min, followed by incubation in 50 °C water for 20 min [102]. After centrifugation at 10,000× *g* for 5 min, the supernatant was filtered through a 0.22 µm pore-size polyethersulfone filter (Sartorius, Surrey, UK), and the filtrate was used for IMCs determination. An Abraxis Microcystin Kit (520011) was used to determine IMCs and EMCs concentrations following the manufacturer’s instructions. The medium was used as blank. All measurements for MCs were done in duplicate, according to the instructions.

### 5.7. Extracellular Polymeric Substances Determination

Soluble EPS (sEPS) and bound EPS (bEPS) concentrations were determined for protein and carbohydrate content, according to the method described elsewhere [103,104]. Briefly, 30 mL of cyanobacteria was cultured as described above for 15 days. After centrifugation at 11,550× *g* for 15 min, sEPS and bEPS were determined in the supernatant and pellet, respectively. To obtain bEPS, the pellet was resuspended in 5 mL distilled water and incubated at 45 °C for 4 h. The mixture was centrifuged at 11,550× *g* for 15 min, and the supernatant containing bEPS was collected. The protein and carbohydrate concentrations of the sEPS and bEPS were quantified by Lowry [105] and phenolsulfuric [106] assays, respectively. The determination was done in triplicate.

Data obtained were analyzed by ANOVA with the post hoc Tukey test using SPSS statistical software version 17.0 for Windows EDU to investigate the significance of EPS content and composition at *p* ≤ 0.05.

### 5.8. Scum Formation

Scum formation was determined by observing cell migration to the water surface. Scum formation pictures were illustrated elsewhere [13,53]. Cyanobacteria culture was prepared for 7 days, as previously described. After centrifugation at 10,000× *g* for 5 min, the pellet was resuspended with 5 mL of 8× concentrated BG-11 medium. The OD_580_ of the culture was adjusted to 1, followed by incubation under the constant fluorescent light of 20 µmol photons m^−2^/s. Scum formation was observed daily for 7 days with the naked eye.

### 5.9. Dynamic of MCs Production and Growth 

*Microcystis* SG03 was cultured in 500 mL BG-11 medium under the above conditions for 30 days. Thirty-one milliliters was collected every 5 days during the cultivation. To measure cyanobacterial growth, cell numbers from 10 µL of each sample were counted under a microscope using a hemocytometer. IMCs and EMCs were determined, as previously described. The experiment was conducted in independent duplicate.

### 5.10. Analyses of Cyanotoxins

*Microcystis* SG03 was cultured in 300 mL BG-11 medium for 10 days, as described above. After centrifugation at × *g* for 5 min, the pellet was resuspended in 5 mL 50% methanol–water solution. The IMCs were extracted using the same method, as previously described. Cyanotoxins were analyzed by a liquid chromatograph-quadrupole time-of-flight mass spectrometer (LC-QTOF MS) (G6545A, Agilent Technologies, CA, USA) [107] equipped with a Zorbax Eclipse plus C18 Rapid Resolution column HD (150 mm length × 2.1 mm inner diameter and particle size 1.8 µm). The column was held at 40 °C with gradient elution: high-purity water containing 0.1% formic acid (solvent A) and 11.5% acetonitrile solution (solvent B). The elution steps were: (1) 70% A and 30% B for 2 min; (2) 5% A and 95% B for 14 min; (3) 70% A and 30% B for 14.20 min; and (4) 70% A and 30% B for 20 min at a flow rate of 0.2 mL/min. The UHPLC system was connected with a quadrupole time-of-flight mass spectrometer, equipped with a Dual Agilent Jet Stream Electrospray Ionization (Dual AJS ESI). The positive electrospray ionization parameters were as follows: gas temp, 325 °C; gas flow, 13 L/min; nebulizer, 35 psig; sheath gas temp., 275 °C; capillary voltage (VCap), 4000 V; nozzle voltage, 2000 V: fragmentor, 175 V: skimmer, 65 V; octopole RF peak, 750 V; mass ranges were set at 100–1500 *m/z* for the TOF-MS scan and the MS/MS experiments. In the auto MS/MS experiment, the collision energies were set at 10, 20, and 40 V. Continuous internal calibration was performed during analyses by using the signals at *m*/*z* 121.0509 (protonated purine) and *m*/*z* 922.0098 (protonated hexakis (1 H,1 H,3H-tetrafluoropropoxy) phosphazine or HP-921) in positive ionization. Acquisition and data processing were achieved using MassHunter WorkStation LC/MS Data Acquisition software and MassHunter WorkStation Qualitative Analysis Workflows software (version B.08.00, Agilent Technologies, CA, USA), respectively. Identification of the cyanotoxins was based on MS/MS standard: Cayman Chemical Item Number 10445 and available microcystin database Toxinmasslist_com_v15b [62].

## Figures and Tables

**Table 1:** Water quality results.

Water Characteristics	Songkhla Lake, Songkhla, Thailand			Thale Noi, Pattalung, Thailand	Hat Yai Municipal Wastewater Treatment Plant (HMWTP)
BOD (mg/L)	4.62	4.62	Below LOD *	3.63	5.4
COD (mg/L)	46	50	28	35	37.6
TP (mg/L)	0.11	0.32	0.08	0.11	0.24
TKN (mg/L)	1.15	1.1	0.78	0.69	0.58
DO (mg/L)	4.37	1.82	4.58	7.02	5.8
Temperature (°C)	32	32	31	29	30
pH	8.34	8.1	7.23	7.38	6.7
Salinity (g/L)	0.3	0.3	0.1	0.1	0.1
Coordinates	7°47′335.9″ N 100°15′28.0″ E	7°4610.0″ N 100°18′08.8″ E	7°41′11.0″ N 100°12′31.1″ E	7°46′40.6″ N 100°07′22.1″ E	7°46′40.6″ N 100°07′22.1″ E
Isolates	SF11	SG03, SG10, SG11, SG12	SH12, SH13	PB07	WOT01, WIN01

* 1.56 mg/L biochemical oxygen demand (BOD), chemical oxygen demand (COD), total phosphorus (TP), total Kjeldahl nitrogen (TKN), and dissolved oxygen (DO).

**Table 2:** Genotypic and phenotypic characteristics of *Microcystis* spp.

*Microcystis* Sp.	Genotypes			Phenotypes		
	*mcyA^a^*	*mcyA^b^*	*stxtA*	The Specific Growth Rate *, µ (Day^−1^)	Chlorophyll a (Day 15, µg/mL)	MCs Production (Day 15, µg/mL)
SF11	-	+	-	0.291	3.386	194
SG03	+	+	+	0.312	4.139	314
PB07	-	+	-	0.332	3.514	188
SG10	+	+	-	0.312	4.182	210
SG11	+	-	+	0.312	4.024	306
SG12	+	+	+	0.329	4.37	281
SH12	+	+	-	0.211	3.889	43
SH13	-	+	-	0.209	4.173	142
WOT01	-	+	+	0.271	3.982	279
WIN01	-	+	+	0.261	4	273

* The specific growth rate was calculated based on cell number.

**Table 3:** Identification of cyanotoxin detected by LC-QTOF MS analysis in *Microcystis* strains SG03 isolated from SLB, their retention times (Rt), observed *m/z* values, and mass accuracy tolerances (Diff, ppm).

	Compound Name	Neutral Formular	Confidence	Rt (min)	*m/z*	Mass	Diff. (ppm)
1	Decarbamoylsaxitoxin	C_9_H_16_N6O_3_	Confirmed	6.431	257.136	256.1286	−0.63
2	[Gly^1^,D-Asp^3^,(EtOH)Mdhb^7^]MC-Y(H2)Y(OMe)	C_57_H_77_N_7_O_16_	Tentative	4.132	558.778	1115.5409	1.56
3	[L-Ser^1^,D-Asp^3^]MC-LY(OMe)	C_52_H_71_N_7_O_15_	Tentative	4.926	517.7576	1033.5005	0.29
4	[DMAdda^5^,Mglu^6^,Mala^7^]MC-Y(H4)A	C_49_H_71_N_7_O_13_	Tentative	4.929	483.764	965.5125	−1.17

## Data Availability

All relevant data are within the paper.
